# First person – Maika S. Deffieu

**DOI:** 10.1242/bio.042499

**Published:** 2019-03-15

## Abstract

First Person is a series of interviews with the first authors of a selection of papers published in Biology Open, helping early-career researchers promote themselves alongside their papers. Maika S. Deffieu is first author on ‘The Toxoplasma dense granule protein TgGRA3 interacts with host Golgi and dysregulates anterograde transport’, published in BIO. Maika conducted the research described in this article while a postdoctoral researcher in Stanislas Tomavo's lab at Pasteur Institute, Lille, France. She is now a postdoctoral researcher in the lab of Raphael Gaudin at IRIM CNRS, Montpellier, France, investigating intracellular trafficking and particularly how micro-organisms divert the host trafficking pathways to survive.


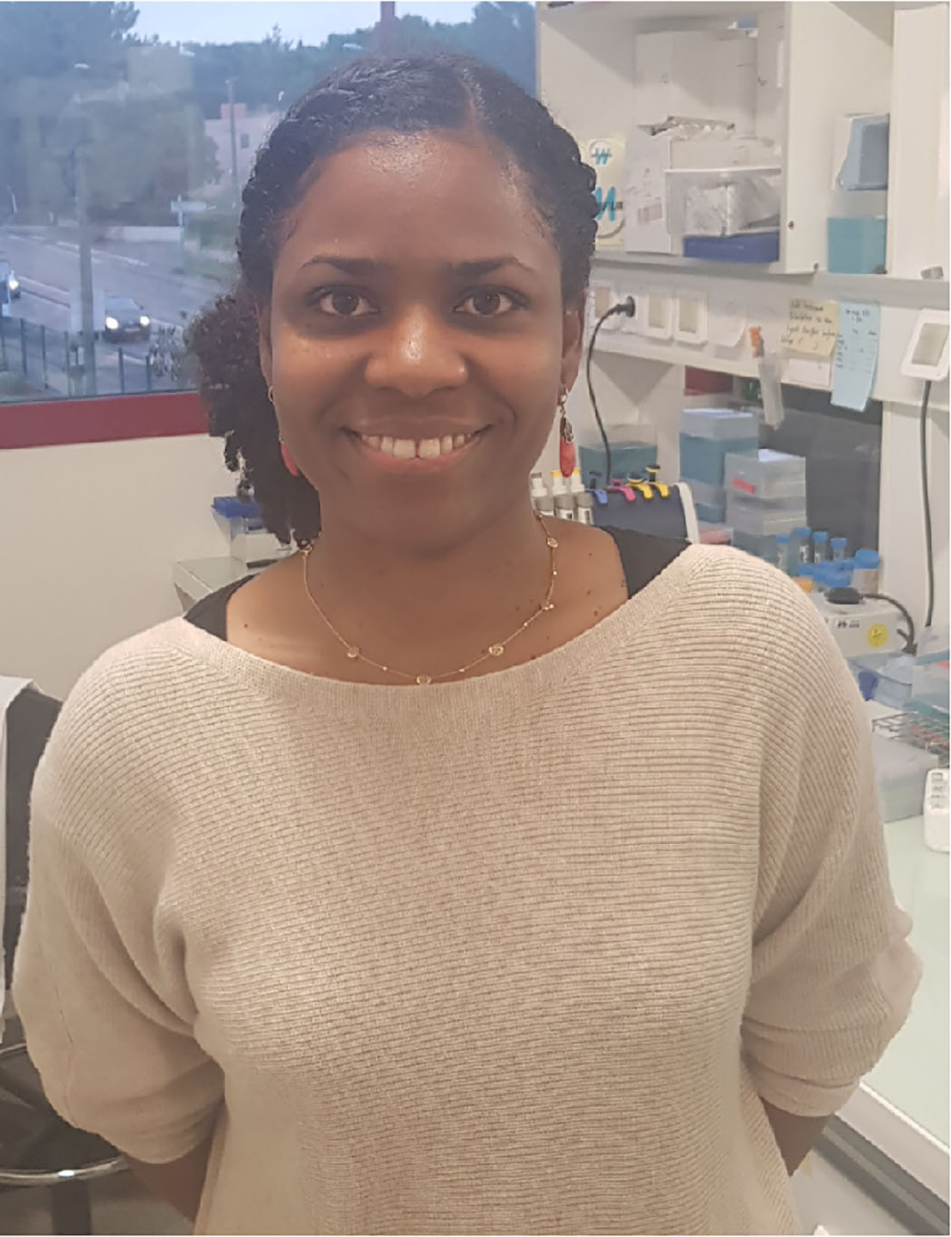


**Maika S. Deffieu**

**What is your scientific background and the general focus of your lab?**

My scientific background is in intracellular trafficking. My PhD was completed at the University of Bordeaux, France, under the mentorship of Dr Nadine Camougrand. During this time, I investigated the signaling pathways inducing mitophagy in yeast *Saccharomyces cerevisiae*. For my postdoctoral studies, I then moved to Stanford University in California to the laboratory of Prof. Suzanne Pfeffer, to study cholesterol export from endosomes in mammalian cells. After which I moved back to France to work in Dr Stanislas Tomavo's laboratory where I investigated the molecular mechanism triggering host organelles recruitment by the parasite *Toxoplasma gondii*. Now, I am working in the team of Dr Raphael Gaudin at the IRIM CNRS institute, France. I am working on deciphering the host cell trafficking pathways used by viruses during their cellular entry.

**How would you explain the main findings of your paper to non-scientific family and friends?**

*Toxoplasma gondii* is a parasite responsible for toxoplasmosis. Toxoplasmosis is a disease that can be dangerous for pregnant women. It can trigger miscarriages the first month of infection, or cause abnormalities in the development of the fetus when the infection happens after the fourth month of pregnancy. In order to infect a person and grow inside the body, the parasite needs to be able to exploit all the tools it can to stay inside the cell, multiply in several other parasites and hide from our immune system. In our research, we tried to look for the strategies used by *Toxoplasma* to modify the cell to its advantage. Understanding this process will help researchers to develop better therapeutic drugs that will stop the parasite progression inside the human body. In our work, we discovered that the parasite uses special proteins to target and consume the cell components. Although we gave a hint to a potential mechanism used by the parasite to manipulate the cell, more elements are still needed to completely understand *Toxoplasma*’s life inside the human body.

**What are the potential implications of these results for your field of research?**

The results indicated that parasites can use a variety of different proteins to be able to scavenge host organelles. We indicated that dense granule proteins such as TgGRA3 can have dual functions. They can mediate host organelles entry, as well as divert the host trafficking pathways. The panel of proteins that we identified could hopefully be used by the community to discover more on how *Toxoplasma* can attract the host Golgi or other organelles. Our protein candidates could also be used to identify new host protein partners in the future. By understanding these molecular mechanisms better, we could be able to specifically target parasite proteins without affecting the host physiology.

“By understanding these molecular mechanisms better, we could be able to specifically target parasite proteins without affecting the host physiology.”

**What has surprised you the most while conducting your research?**

I was surprised to observe how fast the parasite can induce a compensatory mechanism to survive inside the cell. It indicated that there is still a broad field of research ahead of us to understand the parasite's biology.

**Figure:**
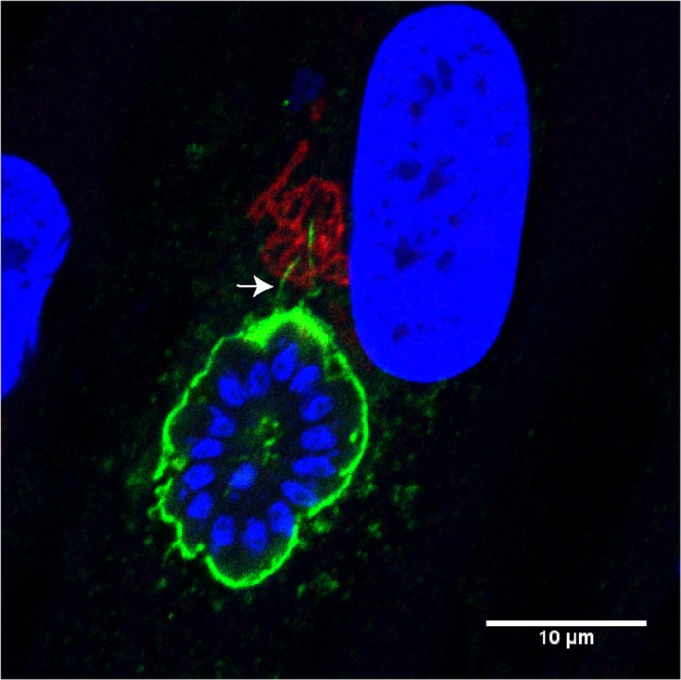
The parasitophorous vacuole of *Toxoplasma gondii* sends filaments (green, white arrow) directed toward the host Golgi (red)

**What, in your opinion, are some of the greatest achievements in your field and how has this influenced your research?**

For me most of the achievements carried out in membrane trafficking influenced my research. Especially the use of tools combining cell biology and biochemistry to solve the mysteries hidden behind the function of a protein. I was always impressed by the complexity of the different molecular mechanisms used by the cell to properly function. Since my PhD I discovered that the functional state of mitochondria can affect their morphology (fusion or fission), or that vesicular trafficking is important for compartments segregation. Investigations and discoveries of all of these different protein complexes, and how they can maintain the proper balance of the cell, had a big impact on how I envision science.

**What changes do you think could improve the professional lives of early-career scientists?**

I consider science as a passion. There are many people who want to continue pursuing this career, but are stopped by the limited number of permanent positions. Moreover, we can also be confronted by the short duration of our contracts that are not compatible with the completion of our projects, considering the time needed to publish our work. I think increasing the number of permanent positions in academia, or having more fellowship programs to extend the work of postdoctoral researchers would be important to improve the professional lives of early-career scientists.

**What's next for you?**

I am currently working on combining intracellular trafficking and virus dynamic. In the future, I will look for permanent positions in academic science. My goal is to keep doing what I like to do best, discovering new things!
